# The plastome sequence of Ulleung Rowan, *Sorbus ulleungensis* (Rosaceae), a new endemic species on Ulleung Island, Korea

**DOI:** 10.1080/23802359.2018.1443042

**Published:** 2018-02-24

**Authors:** Hee-Young Gil, Seung-Chul Kim

**Affiliations:** Department of Biological Sciences, Sungkyunkwan University, Suwon, Republic of Korea

**Keywords:** Plastome, *Sorbus ulleungensis*, Ulleung Rowan, Ulleung Island, Korean endemic

## Abstract

The complete chloroplast genome sequence of *Sorbus ulleungensis*, a recently described endemic species to Ulleung Island of Korea, was determined. The genome size was 159,632 bp in length with 36.5% GC content. It included a pair of inverted repeats (IRa and IRb) of 26,402 bp, which were separated by small single copy (SSC: 18,824 bp) and large single copy (LSC: 88,003 bp) regions. The cp genome contained 111 genes, including 78 protein coding genes, 29 tRNA genes, and four rRNA genes. Phylogenetic analysis of the combined 78 protein coding genes and four rRNA genes showed that *S. ulleungensis* was most closely related to *Pyrus pyrifolia*.

*Sorbus* L. *sensu stricto* (Rosaceae) comprises approximately 90 species and occurs widely throughout northern hemisphere (Lu and Spongberg 2003; McAllister 2005). A dense corymbs of creamy white flowers in spring followed by red, white, pink, orange or yellow foliages, and fruits in fall make this genus very attractive ornamental trees around the world. *Sorbus ulleungensis* Chin S. Chang, was recently described as a new endemic species on Ulleung Island, Korea (Chang and Gil 2014). It has been assigned originally to *Sorbus commixta* based on the morphological similarities, but the morphometric analysis of vegetative and reproductive traits supported taxonomic distinction between the two taxa (Chang and Gil 2014). This species possesses great value as garden trees as well as edible resources for Ulleung Island local people (Nitzelius 1989; Ong et al. 2016).

The wild individual was collected from Ulleung Island, Korea (voucher specimen SKK018558; deposited in the herbarium SKK). Total DNA was extracted from fresh leaves using the DNeasy Plant Mini Kit (Qiagen, Carlsbad, CA). Library preparation and genomic sequencing on the Illumina Miseq platform were conducted by the Macrogen (Macrogen Inc., Seoul, South Korea). The chloroplast genome was assembled using SPAdes 2.425 and CLC Genomics Workbench v.5.5.1 (CLC Bio, Aarhus, Denmark). Then, all the contigs were aligned to the reference chloroplast genome of *Sorbus torminalis* (Ulaszewski et al. 2017) using BLAST (NCBI BLAST v2.2.31) search. The chloroplast genome of *S. ulleungensis* was annotated using Dual Organellar GenoMe Annotator (DOGMA) (Wyman et al. 2004). The tRNA regions were confirmed using tRNAscan-SE with default setting (Schattner et al. 2005). Annotation of the start/stop codons of protein-coding genes was done using BLASTX, Geneious v.10.2.2. (Biomatters Ltd., Auckland, New Zealand), and then manually corrected for intron/exon boundaries. Maximum likelihood analysis was conducted based on concatenated 78 chloroplast coding genes using IQ-TREE v.1.4.2 (Nguyen et al. 2015) with 1000 bootstrap (BS) replications. The complete chloroplast genome of *S. ulleungensis* (GenBank accession MF695711) was 159,632 bp with 36.5% GC content and contained two inverted repeat regions (IRa and IRb) of 26,402 bp, separating a large single copy (LSC) region of 88,003 bp and a small single copy (SSC) region of 18,824 bp. The plastome contained 111 genes, including 78 protein-coding genes, 29 tRNA genes, and four rRNA genes. Eighteen genes, including seven tRNA genes and four rRNA genes, were duplicated in the IR regions. The structure, gene order, gene content, and GC content of the *S. ulleungensis* were similar to those of species of tribe Maleae chloroplast genomes (Bao et al. 2016; Ulaszewski et al. 2017). The phylogeny of two subfamilies (Amygdaloideae and Rosoideae) and four major tribes of Amygdaloideae of Rosaceae is shown in Figure 1. Tribe Maleae of subfamily Amygdaloideae were monophyletic (100% BS, Figure 1). However, *Sorbus sensu lato* was not monophyletic as suggested by Lo and Donoghue (2012). *Sorbus ulleungensis* was most closely related to *Pyrus pyrifolia*. A further detailed work is necessary to elucidate phylogenetic relationships among major lineages of highly complex and specious genus *Sorbus*.

**Figure 1.**
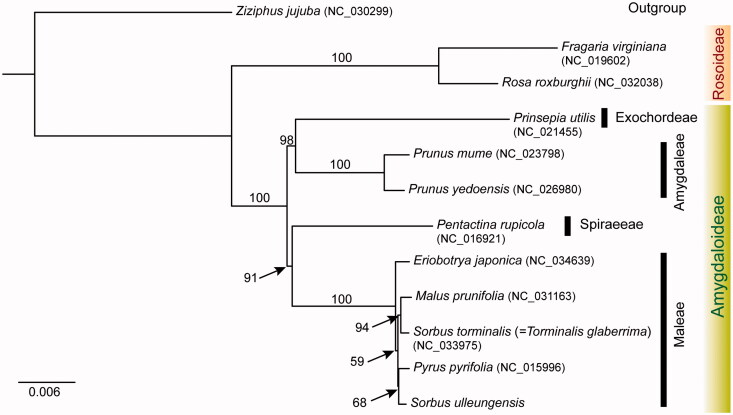
Maximum-likelihood tree based on 78 protein-coding and four rRNA genes from 11 representative species of Rosaceae. *Ziziphus jujuba* (Rhamnaceae) was used as an outgroup and the bootstrap support values are shown at the branches.
